# Effects of Aging on Genioglossus Motor Units in Humans

**DOI:** 10.1371/journal.pone.0104572

**Published:** 2014-08-11

**Authors:** Julian P. Saboisky, Daniel W. Stashuk, Andrew Hamilton-Wright, John Trinder, Sanjeev Nandedkar, Atul Malhotra

**Affiliations:** 1 Division of Sleep Medicine, Brigham and Women’s Hospital, and Harvard Medical School, Boston, Massachusetts, United States of America; 2 Neuroscience Research Australia, Sydney, New South Wales, Australia; 3 Prince of Wales Clinical School, Faculty of Medicine University of New South Wales, Sydney, New South Wales, Australia; 4 Department of Systems Design Engineering, University of Waterloo, Waterloo, Canada; 5 Mathematics and Computer Science, Mount Allison University, Sackville, New Brunswick, Canada; 6 School of Psychological Sciences, University of Melbourne, Melbourne, Victoria, Australia; 7 Natus Medical Inc, Middleton, Wisconsin, United States of America; 8 University of California San Diego, La Jolla, California, United States of America; University of Texas Health Science Center at San Antonio, Research Imaging Institute, United States of America

## Abstract

The genioglossus is a major upper airway dilator muscle thought to be important in obstructive sleep apnea pathogenesis. Aging is a risk factor for obstructive sleep apnea although the mechanisms are unclear and the effects of aging on motor unit remodeled in the genioglossus remains unknown. To assess possible changes associated with aging we compared quantitative parameters related to motor unit potential morphology derived from EMG signals in a sample of older (n = 11; >55 years) *versus* younger (n = 29; <55 years) adults. All data were recorded during quiet breathing with the subjects awake. Diagnostic sleep studies (Apnea Hypopnea Index) confirmed the presence or absence of obstructive sleep apnea. Genioglossus EMG signals were analyzed offline by automated software (DQEMG), which estimated a MUP template from each extracted motor unit potential train (MUPT) for both the selective concentric needle and concentric needle macro (CNMACRO) recorded EMG signals. 2074 MUPTs from 40 subjects (mean±95% CI; older AHI 19.6±9.9 events/hr *versus* younger AHI 30.1±6.1 events/hr) were extracted. MUPs detected in older adults were 32% longer in duration (14.7±0.5 ms *versus* 11.1±0.2 ms; *P*  =  0.05), with similar amplitudes (395.2±25.1 µV *versus* 394.6±13.7 µV). Amplitudes of CNMACRO MUPs detected in older adults were larger by 22% (62.7±6.5 µV *versus* 51.3±3.0 µV; *P*<0.05), with areas 24% larger (160.6±18.6 µV.ms *versus* 130.0±7.4 µV.ms; *P*<0.05) than those detected in younger adults. These results confirm that remodeled motor units are present in the genioglossus muscle of individuals above 55 years, which may have implications for OSA pathogenesis and aging related upper airway collapsibility.

## Introduction

Obstructive sleep apnea is a common disorder that increases in prevalence with age, although the mechanisms are unclear. The genioglossus is a major upper airway dilator muscle whose activity is thought to be representative of muscles critical for maintaining pharyngeal patency. Thus, research into the motor control of the genioglossus is likely to provide insights into sleep apnea pathogenesis. Motor unit potential (MUP) analysis provides insight into the normal function of skeletal muscle and aids in the assessment of neuromuscular disorders [1]. For example, skeletal muscle remodeling is associated with physiological factors that can change the characteristics of MUPs [2]–[4]. MUPs with increased durations can be detected in many skeletal muscles, reflected as remodeled motor units as a result of denervation, collateral sprouting and reinnervation [5]–[7].

Anatomically the genioglossus muscle is one of the largest extrinsic muscles of the tongue [8]–[10]. The hypoglossal nerve branches that innervate the genioglossus muscle are much denser in humans compared to other species [11]–[15], likely reflecting small motor unit territories required for the high level of fine motor control required for speech. The complex innervation of the muscles of the tongue may indicate they are less prone to aging effects than is seen in other skeletal muscles [16]. Structural remodeling changes previously reported in the tongue musculature of obstructive sleep apnea patients [17] may not be characterized by a proximal weakness, such as, overt dysphagia, but, may nevertheless predispose the pharyngeal airway to collapse with increasing age.

The activity of the human genioglossus is complex, with activity in phase with both inspiration and expiration [18]–[21]. Anatomically the position of the genioglossus muscle and its role in dilating the airway is of great interest in understanding the pathogenesis of obstructive sleep apnea [17], [22]–[24]. The tongue also plays a critical role in swallowing and speech pathologies [25]; therefore, understanding how the neuromuscular innervation may be remodeled with aging is clinically important and may provide insight for therapy or treatment based on age.

Changes that occur within the pharyngeal musculature with aging remain incompletely understood. Despite the fact that the genioglossus muscle is known to contain a high proportion of Type II muscle fibers and aging effects are pronounced for Type II fibers, [26], [27], we recently found no evidence for increased MUP durations associated with age in humans [17]. While some investigators have found a continuous increase of MUP durations in skeletal muscles from 1 year of life up to 80 years [28] other investigators have demonstrated that the aging effect on MUP duration occurs predominantly after 55 years of age [29]. Previously, we reported neural injury associated with obstructive sleep apnea patients, observed by an increase in the durations of MUPs of the genioglossus muscle of humans [17], [22]. However, these investigations were not specifically targeted to investigate aging, as we included primarily younger adults.

Limited information exists about changes in the morphology of MUPs with aging. Based on the aging literature compensatory adaptation with muscle fiber hypertrophy or neurogenic changes such as collateral sprouting of motor axons may occur indicating a remodelling of the motor units [2], [30], [31]. We aimed to investigate the effect of aging, in genioglossus muscle, with a range of conventional and specialized electromyographic techniques to obtain features of EMG signals that relate to one or more aspects of normal and pathologic function. Thus, we measured the activity from the genioglossus in younger and older adults (both with and without obstructive sleep apnea) while they were awake and breathing quietly. Based on the MUP literature we hypothesized that MUPs detected in older adults (>55 years) would show signs for greater degrees of reinnervation (collateral axonal sprouting).

## Methods

The present investigation was based on new analyses of motor units obtained during awake, quiet breathing from a previous experiment [17], together with newly acquired data from nine older subjects. The previous investigation was designed to confirm neurogenic changes in patients with obstructive sleep apnea *versus* healthy control participants. This current reanalysis allowed us to compare a large number of MUPs in a group of participants spanning a large age range who were all screened for obstructive sleep apnea. Demographic details of the older and younger group are given in Table 1. Control subjects were defined with an AHI of less than 10 events/hr. Newly acquired data included: four controls [65±2.8 years, age range: 61–67 years, apnea hypopnea index (AHI): 4.5±2.1 events/hr, AHI range: 0.6–9.3 events/hr] and five OSA [67±5.6 years, age range: 58–74 years, AHI: 36.3±12.1 events/hr, AHI range: 24–56 events/hr]. The demographic details for a detailed sub-analysis on control subjects are given in Table 2. All subjects gave written, informed consent before participation in this study, which had been approved by the Partners’ Human Research Committee and conformed to the Declaration of Helsinki.

**Table 1:** Subject demographics for study populations.

Group subject characteristics	Older	Younger
**Subjects**	11	29
**Age (years)**	65.2±4.6	36.8±2.9
	(Age range: 74–58)	(Age range: 53–19)
**Female/male**	2/9	8/21
**Apnea hypopnea index (events per hour of sleep)**	19.6±9.9	30.1±6.1
	(AHI range: 56–0.6)	(AHI range: 93.6–0.6)
**Height (cm)**	174.2±6.1	173.7±4.0
**Weight (kg)**	81.9±11.4	94.0±9.1
**Body mass index (BMI, kg/m^2^)**	27.5±3.1	30.9±2.4*
**Neck size (cm)**	38.9±2.6	40.3±1.5
**Number of units per subject**	49.7±15.0	52.7±7.9
**Inferior margin of geniohyoid muscle (mm)**	15.9±2.6	15.1±1.5
**Inferior margin of genioglossus muscle (mm)**	24.8±2.4	25.3±1.5
**Genioglossus width (mm)**	16.3±0.8	18.0±0.8
**Modified borg scale (0–10)**	2.8±0.8	2.4±0.5

The modified Borg Scale was used to measure the “worst level of pain” experienced by each subject during the recording with 0 =  Nothing at all and 10 =  Maximal. Values are Mean ± CI.

Values are Mean ± CI. *indicates *P*<0.05.

**Table 2:** Demographics for older versus younger control subjects.

Group subject characteristics	Older	Younger
**Subjects**	5	13
**Age (years)**	63.8±3.2	32.5±4.5
	(Age range: 67–59)	(Age range: 42–19)
**Female/male**	¼	6/7
**Apnea hypopnea index (events per hour of sleep)**	5.1±3.3	3.4±1.4
	(AHI range: 9.3–0.6)	(AHI range: 8.7–0.6)
**Height (cm)**	170.4±9.3	169.3±6.7
**Weight (kg)**	72.9±13.1	85.4±15.9
**Body mass index (BMI, kg/m^2^)**	27.3±4.8	29.3±3.9
**Neck size (cm)**	38.1±3.2	38.3±2.6
**Number of units per subject**	61.2±28.7	47.9±9.3
**Inferior margin of geniohyoid muscle (mm)**	15.2±0.4	14.3±0.2
**Inferior margin of genioglossus muscle (mm)**	23.8±0.3	23.6±0.2
**Genioglossus width (mm)**	16.3±0.2	17.7±0.1
**Modified borg scale (0–10)**	3.2±1.1	2.7±0.9

Control subjects were defined with an AHI of less than 10 events/hr.

### GENERAL PROCEDURES

All subjects were initially screened by telephone prior to their arrival at the Brigham and Women’s Hospital Center for Clinical Investigation. Upon arrival each subject underwent thorough medical evaluation to exclude those with a history or physical evidence of major medical (including diabetes) or neurological issues, or sleep disorders other than OSA. One subject was being treated for back pain with gabapentin (subject refrained from taking medication for a week prior to the study), two for depression taking Cymbalta (*Duloxetine, SSRI*), and Citalopram (*SSRI*) and three for hypertension with lisinopril (*angiotensin converting enzyme inhibitor*). Demographic data were recorded for each subject, including height, weight, and age. All subjects were screened for obstructive sleep apnea with a full overnight polysomonography examination.

### RECORDINGS

To determine the depth and location of the needle electrodes in the genioglossus the anatomy of the pharyngeal musculature was examined with ultrasonography (12 L high-frequency linear array transducer, Vivid *i* GE Healthcare Chalfont St. Giles, Bucks, UK) [18], [19], [22], [32]. The distance from the surface of the skin to the inferior margin of the genioglossus and geniohyoid muscles and the lateral width of the genioglossus were recorded using an electronic caliper [32] (see Table 1 for values).

Topical anesthetic cream (Emla, AstraZeneca) was placed on the surface of the skin under the chin for a minimum of 30 minutes. The chin was then thoroughly cleaned using disposable topical antiseptic wipes and a small reference location was drawn 10 mm posterior to genial tubercle in the midline under the chin. With the subjects relaxed and lying supine two concentric needle electrodes (26G, 50 mm, recording area 0.07 mm^2,^ TECA ELITE) were inserted lateral to the midline at 90° to the skin surface and advanced through underlying muscle layers (the mylohyoid and geniohyoid) before entering into the genioglossus (>25.2 mm on average as determined by ultrasound). Maximal depths for the electrodes were marked on the needle cannulae to ensure all recordings were from the genioglossus muscle. The first concentric needle was used as a concentric needle macro electrode (CNMACRO) and was positioned ∼10 mm beyond the genioglossus/geniohyoid aponeurosis where it remained in the same position for the entire recording procedure. The second selective concentric needle electrode was initially positioned to the same depth and in close proximity (∼4 mm) to the CNMACRO electrode. The concentric needle electrode was moved to different locations in the muscle by changing the angle and depth of the electrode following a stable recording at each site. The aim was to sample a minimum of ∼10 independent locations per subject during quiet breathing (held at each site for approximately 30 seconds). A large flexible ground electrode (1180, 3 M Health Care, St. Paul MN) was placed over the right clavicle.

The CNMACRO signals were detected by the cannula of the CNMACRO electrode referenced to a remote surface electrode positioned over the bony mandible (MEDI-TRACE 100 series, Kendall Healthcare, Mansfield, MA) [33]. CNMACRO signals were obtained by bypassing the Sapphire^II^, (TECA, NY) system and recording directly using external amplifiers (15A54 Quad Neuroamplifiers Grass Instruments). The CNMACRO signals were filtered (10 Hz to 6 kHz) and amplified (×10 k). The selective concentric needle signals were detected in a standard manner, filtered (10 Hz to 10 kHz) and displayed with an amplification of 200 µV/division (Sapphire^II^, TECA, NY). Both EMG signals were simultaneously sampled at 25 kHz (Spike2 with 1401 interface, Cambridge Electronic Design, Cambridge, UK), and stored for offline analysis.

During the procedure the subjects were monitored as they breathed through a nasal mask (Gel Mask, Respironics, Murrysville, PA) attached to a heated pneumotachograph (model 3700A, Hans-Rudolph Inc, Kansas City, USA) and a differential pressure transducer (Validyne, Northbridge, CA). End-tidal CO_2_ at the nares (PETCO_2_) was measured through ports in the nasal mask. The calibrated flow signal was integrated to derive volume. Throughout the procedure subjects lay comfortably supine and relaxed, breathed quietly and remained awake. Wakefulness was confirmed by the investigators directly observing the subjects. All the recordings were obtained without the subjects’ experiencing discomfort or pain as measured through a 0–10 modified Borg Scale [22].

### EXTRACTION OF SINGLE MOTOR UNIT ACTIVITY

**Figure 1:**
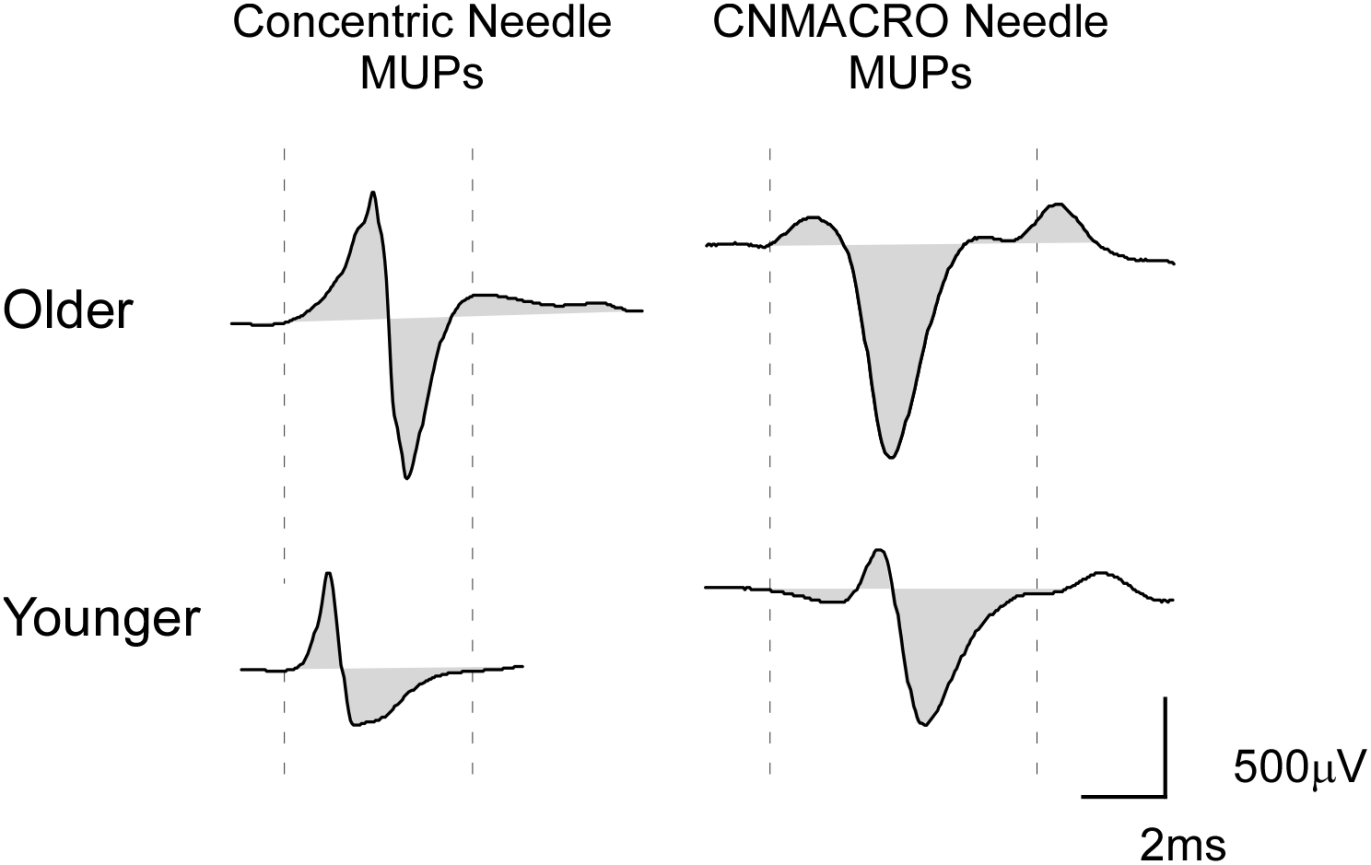
Typical example of genioglossus data. Shown is a typical example of two selective concentric MUP (left) and CNMACRO MUP (right) templates from a recording from an older (top panel; 71 years, AHI 2.8) and younger (lower panel; 38 years, AHI 9.3) subject. Calibrations of the selective concentric needle MUP and CNMACRO MUP, 500 µV and 2 ms. The duration of the MUP as measured between the onset of the first and the offset of the last deviation from the baseline of the MUP are indicated by the vertical dotted lines. The duration is marked relative to the timing of the young MUPs to indicate the lengthening of the MUPs in older subjects.

EMG signals were converted into DQEMG format using customized software (Spike2 scripts). Motor unit potential trains (MUPTs) were extracted from the digitized concentric needle EMG signals off-line using the DQEMG analysis system (version 3.4, revision, 166) [34]–[36] (see Figure 1). During EMG signal decomposition MUPs were detected using absolute detection threshold criteria (slope threshold, 0.3 V/s; amplitude threshold, 50 µV and; time interval, 0.2 ms). For each valid MUPT identified, an average MUP template was extracted. All MUP feature values for all of the subjects were evaluated by a single investigator using established methods [37], that have been previously reported [17]. MUP duration was measured between the onset of the first and the offset of the last deviation from baseline of the MUP. Duration is associated with the quantity of muscle fibers that contribute to the MUP. Amplitude was calculated as the peak-to-peak voltage difference of the MUP. Amplitude is influenced by the diameter and number of muscle fibers closest to the electrode. Area was measured as the sum of the rectified MUP sample values over the duration (in µVs) times the sampling interval (in ms). Area is less susceptible to measurement miscalculation than amplitude and is able to characterize abnormal potentials. The number of MUP phases was defined as the number of baseline crossings minus 1. A turn of a MUP was defined as a change in the direction of the MUP waveform with a magnitude of at least 25 µV. The complexity of a MUP was quantified with a number of indices, including the resulting: area/phase ratio, which is better able to distinguish myopathic MUPs from normal MUPs with greater accuracy than area alone. The size index and area/amplitude ratio [38] are parameters that assist in distinguishing between neurogenic and regular MUPs and control for the effect of needle position [39]. The relative irregularity coefficient [40], [41] encapsulates the complexity of the MUP waveform and is calculated as: RIR = [(S–2A)/2A]×100 (where A is the amplitude, and S is calculated as the sum of the absolute values of the intersample changes seen in the MUP waveform template, i.e., the irregularity in the MUP template). Thus, with partial denervation, denervated muscle fibers are reinnervated by peripheral sprouting thereby the territory the remaining motor units occupy is increased [42]. These new axonal branches conduct impulses with a lower velocity over extended distances, reflected as longer duration and more complex MUP waveforms. The maintenance of muscle size and force generating ability effectively conceals the decreased numbers of surviving motoneurons that have regenerated their axons [43]. All parameter values were manually inspected by a single investigator using DQEMG who was blinded to subject OSA status. For each valid concentric needle MUPT extracted a CNMACRO MUP template was extracted from the raw cannula signal using ensemble averaging. The structures of the concentric needle macro (CNMACRO) MUPs were also measured, using previously established methods [17].

### DATA ANALYSIS

Statistical differences for the dependent MUP feature values were assessed using two-way analyses of variance (ANOVA) to determine the effects of older and younger adults, with the interaction of age with obstructive sleep apnea status (patients and control subjects). Remaining comparisons between older and younger adults were performed using one-way ANOVA. Comparisons utilized the Student-Newman-Keuls post-hoc analysis. As data were not normally distributed a Kruskal-Wallis one way ANOVA on ranks was performed with Dunn’s method post-hoc test applied. The non-parametric statistics are reported in the Figures (SigmaPlot 11). Statistical significance was set at *P*<0.05. Values are given as the mean ± 95% CI.

## Results

### TOTAL ANALYSIS

Electromyographic data were recorded from forty subjects yielding a total of 671 successful unitary recordings from the genioglossus muscle with selective concentric needle and concentric needle macro (CNMACRO) electrodes. The DQEMG algorithms provided a total of 2074 selective concentric needle MUP templates (older 547 *versus* younger 1527), and 1526 CNMACRO MUP templates (older 493 *versus* younger 1033). From these recordings 929 of the motor units sampled were from control subjects (306 older [4.4±0.4 events/hr] *versus* 623 younger [3.6±0.2 events/hr]). On average, the number of MUPTs extracted per recording was lower for older adults compared to younger adults (2.7±0.3 *versus* 3.2±0.2; *P*<0.05, *Dunns Method*). Data underlying the findings reported herein can be located in the File S1. “DQEMG_Data_Aging”.

### MOTOR UNIT POTENTIALS

The compounded effects of aging together with the influence of obstructive sleep apnea effects were investigated with two approaches; utilizing a two-way ANOVA and a detailed sub-analysis (see *Detailed sub-analysis* below).

**Figure 2:**
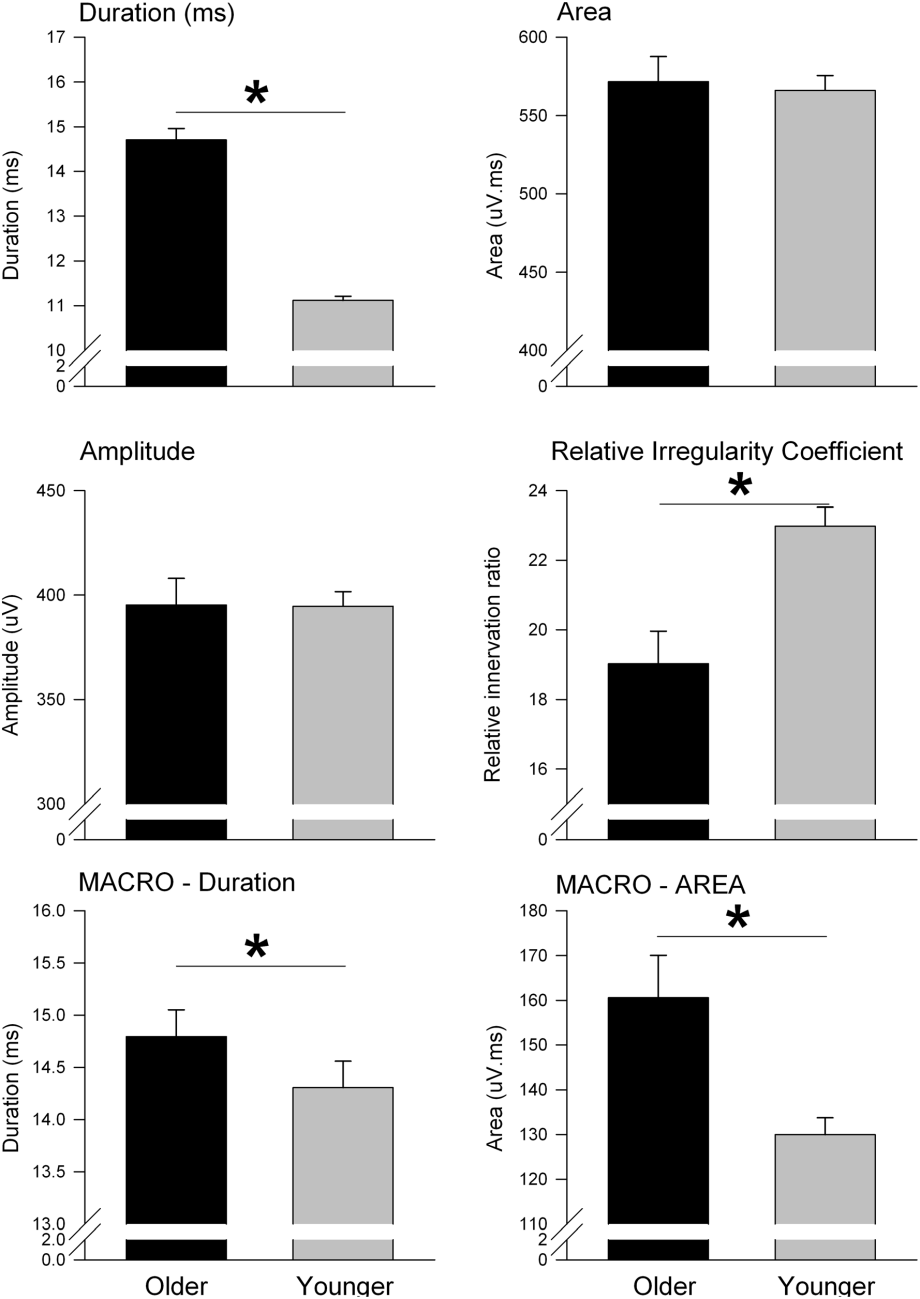
Mean feature values of selective concentric needle MUPs and concentric needle macro (CNMACRO) MUPs. Each panel represents mean values of selected features from all the individual MUPs and CNMACRO MUPs. The solid columns depict values from older subjects and the shaded columns represent values from younger subjects. Significance is given where appropriate in the respective panels.

**Figure 3:**
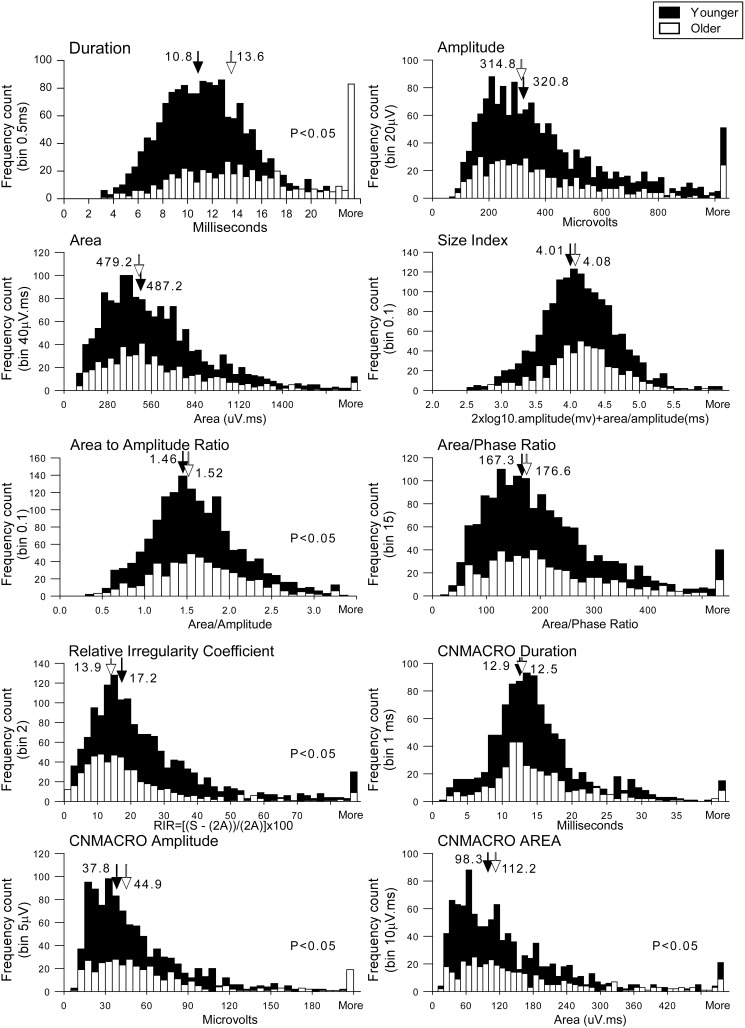
Histogram display with the featured values of selective concentric needle MUPs and concentric needle macro (CNMACRO) MUPs from all 40 subjects. The solid filled columns depict values from younger subjects and the white columns represent values from younger subjects. Medians are indicated by arrows in each panel and in each circumstance the filled arrow indicates the median of younger subjects and the unfilled arrow depicts the median of older subjects. Significance is given where appropriate in the respective panels.

**Figure 4:**
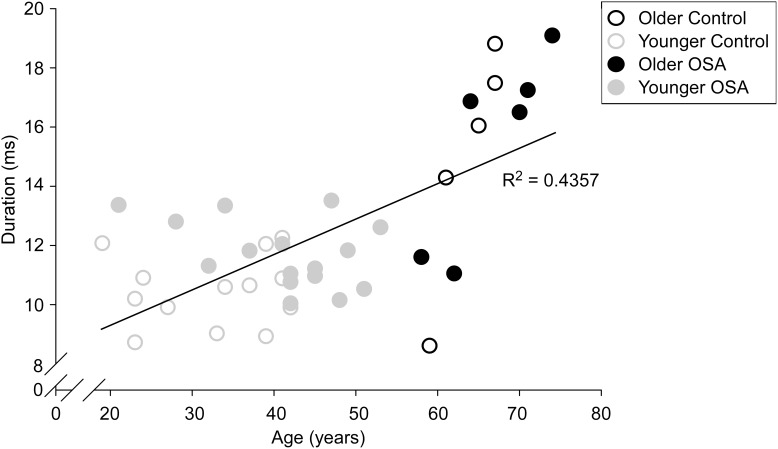
Duration of MUPs plotted versus subject age. Mean MUP duration (ms) versus age (years). The MUPs with longer durations are associated with increased age. The Pearson correlation coefficient was calculated from the mean value of all 40 subjects (r  =  0.660; P  =  0.00000359). The open black circles depict values from older control subjects while solid circles depict values from older obstructive sleep apnea subjects. The open grey circles depict values from younger control subjects while solid circles depict values from younger obstructive sleep apnea subjects.

The selective concentric needle MUPs detected in older adults had 34% longer duration (*P*<0.001), with greater thickness (*P*<0.05), but did not have greater amplitude, area, number of phases or RIR coefficient, than those detected in younger adults (see Figures 2, 3 & 4; Table 3).

**Table 3:** Key results from all 40 subjects.

	Older	Younger
**Number of MUP templates**	547	1527
**Number of MUPTs/recording**	2.7±0.3	3.2±0.2*
**Duration (ms)**	14.8±0.4	11.0±0.2*
**Number of phases**	2.8±0.1	2.9±0.0*
**Number of turns**	3.7±0.2	3.7±0.1
**Amplitude (µV)**	400.3±23.6	393.1±14.3
**Area (µV.ms)**	578.7±31.2	560.4±18.8
**Thickness**	1.6±0.0	1.5±0.0*
**Area/Phase**	208.6±11.0	196.3±6.6
**RIR**	19.0±1.8	22.3±1.1*
**Size index**	4.1±0.0	4.0±0.0
**Number of CNMACRO MUP templates**	383	1033
**MACRO area (µV.ms)**	167.7±14.2	125.1±8.8*
**MACRO amplitude (µV)**	65.4±5.4	49.2±3.4*
**MACRO duration (ms)**	14.9±0.9	14.1±0.5

Values are Mean ± CI. *indicates a significant difference *P*<0.05.

**Figure 5:**
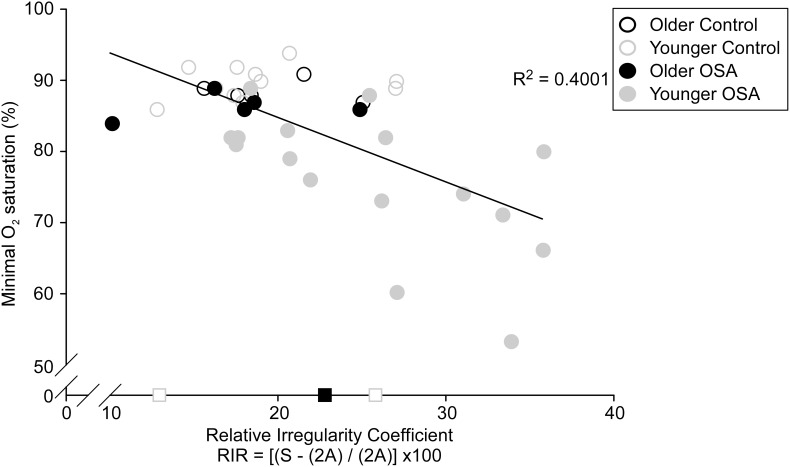
Mean relative irregularity coefficient versus minimal oxygen saturation. More complex motor unit potentials (MUPs), as calculated with the relative irregularity, are associated with minimal oxygen saturation detected from the overnight polysomnography. The Pearson correlation coefficient was calculated from the mean value of 37 subjects (r = 0.633; P = 0.0000267). The three squares on the X-axis represent relative irregularity coefficient data from subjects whose minimal oxygen data were not obtained (not included in the correlation).

For the OSA patients, MUPs with increased amplitudes were detected in older patients compared to younger patients (442.2±35.3 µV *versus* 401.2±18.1 µV; *P*<0.05). However, for the OSA patients, more complex MUPs, as quantified using the RIR coefficient, were detected in younger patients compared to older patients (25.9±1.4 *versus* 18.3±2.7; *P*<0.05; Figure 2). We found that the complexity of the motor unit waveform as calculated with the RIR coefficient to correlate with the minimal oxygen saturations (Figure 5, R^2^ = 0.4, *P*<0.001).

### CONCENTRIC NEEDLE MACRO MOTOR UNIT POTENTIALS

CNMACRO MUPs reflect the overall number of muscle fibers in a motor unit [16]. We estimated a CNMACRO MUP template by ensemble averaging the cannula recorded EMG signal using the discharge times of the respective individual motor unit during normal eupnic breathing. As not all sites that recorded a MUP had an adequately measureable CNMARCO MUP the total number was 1415. The CNMACRO MUPs detected in older adults had larger amplitudes (32%; *P*<0.001), and areas (34%) than those detected in younger adults (*P*<0.001; see Table 3 and Figure 2 & 3).

The CNMARCO MUP area was larger in OSA patients compared to healthy controls by, 37.2% in the younger subjects and 57.6% in the older subjects (both *P*<0.05). In addition, the older subjects had significantly larger CNMARCO MUP areas than the younger subjects within both the OSA and healthy control comparisons (*P*< 0.05).

### DETAILED SUB-ANALYSIS

To determine the effect of aging independent of the presence of obstructive sleep apnea (a known contributor of neurogenic changes in the genioglossus muscle [17]) a sub-analysis was performed on the eighteen control subjects. The older adults (n  =  5) AHI was 5.1±3.3 events/hr *versus* younger adults (n  =  13) 3.4±1.4 events/hr, (range 0.6-9.3 events/hr; Table 2 for demographic properties). This analysis included 306 MUP templates and 228 CNMACRO MUP templates from older adults and 623 MUP templates and 389 CNMACRO MUP templates from younger adults (for results see Table 4). The selective concentric needle MUPs detected in older adults had longer durations (34%, see Figure 4), and greater thickness (14%) compared to those detected in younger adults (*P*<0.05). CNMACRO MUPs detected in the older adults had greater area (23%) and amplitude (25%) compared to those detected in younger adults (*P*<0.05).

**Table 4:** Key results for the 18 control subjects.

	Older	Younger
**Number of MUP templates**	306	623
**Number of MUPTs/recording**	2.9±0.3	3.0±0.3
**Duration (ms)**	14.1±0.7	10.5±0.3*
**Amplitude (µV)**	358.3±29.1	385.1±20.2
**Number of phases**	2.8±0.1	2.8±0.1*
**Number of turns**	3.7±0.2	3.5±0.1
**Area (µV.ms)**	520.3±38.8	529.2±27.7
**Thickness**	1.6±0.1	1.4±0.0*
**Area/Phase**	188.4±13.3	188.8±9.6
**RIR**	19.6±2.2	18.7±1.3
**Size index**	4.0±0.1	4.0±0.0*
**Number of CNMACRO MUP templates**	228	389
**MACRO amplitude (µV)**	51.2±6.2	40.7±3.4*
**MACRO area (µV.ms)**	130.2±15.2	105.5±8.4*
**MACRO duration (ms)**	14.3±1.1	13.2±0.6

## Discussion

This study clearly demonstrates age-related changes in motor unit potentials (MUPs) detected in the genioglossus muscle. We found evidence for remodeling suggesting denervation, collateral sprouting and reinnervation of orphaned muscle fibers leading to increased motor unit size, spatial dispersion of motor unit territories and, size and temporal dispersion of motor unit potential (MUP) components in older adults. The functional consequences of upper airway remodeling remain inconclusive; however, the age related changes are likely to involve an increased predisposition to obstructive sleep apnea (OSA).

Aging is a major factor contributing to the risk of obstructive sleep apnea, although the exact mechanisms as to how aging affects OSA risk remains incompletely understood (for review see: [44]). Multiple investigators have found increased prevalence of sleep apnea in older individuals from cross sectional studies [45]–[47]. Despite a few pathophysiological traits having been explored the proportion of key anatomic and neuro-physiological variables that may indeed contribute to the worsening of OSA are not clearly defined. Some factors linked to OSA, such as the ventilatory control system [48], parasympathetic-nerve activity [49]–[51], arousal threshold [52] and cardiovascular responses to arousal [53], [54] remain stable in older adults. However, there have been a number of factors documented that may predispose individuals to obstructive events and which are age related [55], [56]. Anatomically the volume of the parapharyngeal fat pads has been documented to increase with age [57] independent of overall body fat and neck circumference [58]. However, aging effects on upper airway anatomy are variable in different studies, perhaps reflecting the complexity of sleep apnea pathogenesis [57]–[62]. Furthermore, sensory impairments may be linked to a decrement in the genioglossus reflexes during wakefulness [57], although effects of aging on overall pharyngeal motor control are also complex [63]–[66]. These physiological traits may increase the susceptibility for upper airway collapse that occurs in older adults [52].

Our study supports the hypothesis that older individuals have neurogenic changes in the genioglossus muscle. We found MUPs with increased durations and numbers of phases suggesting that peripheral axons have sprouted to compensate for a loss of motor axons. These data indicate that the presence of ‘neurogenic’ changes is associated with the physiological aging processes in the genioglossus muscle. In addition, as we detected a lower number of motor unit potential trains per recording in older subjects, this finding may indicate fewer motor neurons and thus a lower level of motor unit recruitment in older adults. Importantly, these data were collected during wakefulness. In young adults approximately 50% of inspiratory modulated genioglossus motor units become inactive at sleep onset [67]. While there is no data on older adults, if the proportional fall were the same as in young adults, we speculate that at sleep onset fewer motoneurons would be active in older adults leading to repetitive airway collapse. Interestingly, the more complex MUPs, were detected in younger patients indicating ongoing reinnervation. The smoothed less complex MUPs in the older adults may indicate a reduced level of ongoing, as compared to completed, reinnervation in the older adults.

We sampled motor units using two established electromyographic techniques both selective concentric needle electrodes, and non-selective concentric needle macro (CNMACRO) electrodes. These two methods complement each other such that the fiber density and fiber distribution of the whole cross-section of a motor unit can be explored [68], [69]. While a selective concentric needle MUP with prolonged duration can be an indicator of localized peripheral neuropathy, the size of a CNMACRO MUP is an established parameter related to the number of fibers belonging to its respective motor unit. Specifically, the area of a CNMACRO MUP has been linked to the twitch force generated by its motor unit [33], [70]. CNMACRO MUPs therefore can provide unique insight into the contribution of a motor unit to the force produced by a muscle. Our evidence of increased amplitude and area of CNMACRO MUPs demonstrates that older adults may have increased force generation per motor unit activation. Accordingly, our results suggest a greater percentage of muscle fibers will be recruited with each depolarization of a motoneuron for older adults; thereby, changes are partially concealed through the increased force produced of reinnervated orphaned fibers [71]. This finding suggests that the upper airway in older adults may be compromised due to an overall loss of motoneurons. The airway is therefore, possibly more likely to collapse due to fewer motor units available to be recruited during quiet breathing (despite the compensatory changes in force output of remodeled units). Overall, a smaller reserve of motor units remains available for dilating the airway due to the remodeling processes within the muscle.

Our study has a number of strengths. In contrast to prior studies we used ultrasound guidance to ensure electrodes were placed in the genioglossus [72], [73]. In addition, we studied the genioglossus muscle during normal eupnic breathing, as opposed to studies using volitional output that may target different non-respiratory motoneurons [72], [73]. Thus, these data are of high relevance to sleep apnea and its marked increase in prevalence with aging. Finally, we assessed a large sample of motor units in extensive analyses of a large number of patients and therefore believe this is the most rigorous electrophysiological evaluation of the upper airway muscles in older humans to date.

Despite these strengths, we acknowledge a number of weaknesses. First, our groups were incompletely matched for gender, and body mass index. However, the electrophysiological methods used are robust and well validated [36], [39], [74], [75] and less susceptible to sampling bias than biopsy in humans. Unfortunately, the cross-sectional design of this study means that it is not possible to identify the causative nature of this remodeling. Our newly acquired data were blinded to the results of the sleep study and as a result many of our newly acquired older subjects did have mild sleep apnea; therefore, the results may be confounded by this variable. However, as clearly observable in the results of the sub-analysis reported in Table 4, independent of sleep apnea there is an age related increase in a number of key parameters. Finally, the data in this study showed a very clear, highly significant main effect of age in the two-way ANOVA. Therefore, despite these acknowledged limitations, we believe our findings provide an important advance in the literature.

In summary, the results of this study indicate that neurogenic changes occur in the genioglossus muscle of older adults. While it is unlikely a single mechanism can explain all the data concerning the alterations in the MUPs observed, we believe these changes reflect the ongoing influence of age [76]. The results indicate, structural neurogenic changes are present in older adults.

## Supporting Information

File S1 **“DQEMG_Data_Aging”.**
(XLSX)

## References

[1] Buchthal F (1977) Electrophysiological signs of myopathy as related with muscle biopsy. Acta Neurologica 32: 1–29. 855688

[2] Takeda N, Thomas GR, Ludlow CL (2000) Aging effects on motor units in the human thyroarytenoid muscle. The Laryngoscope 110: 1018–1025. 10852524 10.1097/00005537-200006000-00025

[3] Buchthal F, Guld C, Rosenfalck P (1954) Action potential parameters in normal human muscle and their dependence on physical variables. Acta Physiologica Scandinavica 32: 200–218. 13228109 10.1111/j.1748-1716.1954.tb01167.x

[4] Tishler PV, Larkin EK, Schluchter MD, Redline S (2003) Incidence of sleep-disordered breathing in an urban adult population: the relative importance of risk factors in the development of sleep-disordered breathing. JAMA 289: 2230–2237. 12734134 10.1001/jama.289.17.2230

[5] Nandedkar SD, Sanders DB, Stålberg EV (1988) EMG of reinnervated motor units: a simulation study. Electroencephalography Clininical Neurophysiology 70: 177–184. 10.1016/0013-4694(88)90117-4 2456195

[6] Svanborg E (2005) Impact of obstructive apnea syndrome on upper airway respiratory muscles. Respiratory Physiology and Neurobiology 147: 263–272. 16054444 10.1016/j.resp.2005.06.012

[7] Fuglsang-Frederiksen A, Scheel U, Buchthal F (1977) Diagnostic yield of the analysis of the pattern of electrical activity of muscle and of individual motor unit potentials in neurogenic involvement. Journal of Neurology, Neurosurgery & Psychiatry 40: 544–554. 10.1136/jnnp.40.6.544 PMC492760 903769

[8] Abd-El- Malek S (1938) A contribution to the study of the movements of the tongue in animals, with special reference to the cat. Journal of Anatomy 73: 15–31. 17104743 PMC1252528

[9] Takemoto H (2001) Morphological analyses of the human tongue musculature for three-dimensional modeling. Journal of Speech Language and Hearing Research 44: 95–107. 10.1044/1092-4388(2001/009) 11218113

[10] Anderson RJ (1881) The morphology of the muscles of the tongue and pharynx. Journal of Anatomy and Physiology 15: 382–391. PMC1310003 17231393

[11] McClung JR, Goldberg SJ (2000) Functional anatomy of the hypoglossal innervated muscles of the rat tongue: a model for elongation and protrusion of the mammalian tongue. Anatomical Record 260: 378–386. 11074403 10.1002/1097-0185(20001201)260:4<378::AID-AR70>3.0.CO;2-A

[12] Mu L, Sanders I (2000) Neuromuscular specializations of the pharyngeal dilator muscles: II. Compartmentalization of the canine genioglossus muscle. Anatomical Record 260: 308–325. 11066041 10.1002/1097-0185(20001101)260:3<308::AID-AR70>3.0.CO;2-N

[13] Mu L, Sanders I (2010) Human tongue neuroanatomy: Nerve supply and motor endplates. Clinical Anatomy 23: 777–791. 20607833 10.1002/ca.21011 PMC2955167

[14] Fitzgerald MJ, Law ME (1958) The peripheral connexions between the lingual and hypoglossal nerves. Journal of Anatomy 92: 178–188. 13525233 PMC1249692

[15] Curto FS Jr, Suarez F, Kornblut AD (1980) The extracranial hypoglossal nerve: 112 cadaver dissection. Ear Nose and Throat Journal 59: 94–99. 7371568

[16] Stålberg E (1982) Macroelectromyography in reinnervation. Muscle & Nerve 5: S135–138. 6302490

[17] Saboisky JP, Stashuk DW, Hamilton-Wright A, Carusona AL, Campana LM, et al (2012) Neurogenic changes in the upper airway of obstructive sleep apnea patients. American Journal of Respiratory and Critical Care Medicine 185: 322–329. 22016445 10.1164/rccm.201106-1058OC PMC3297112

[18] Saboisky JP, Butler JE, Fogel RB, Taylor JL, Trinder JA, et al (2006) Tonic and phasic respiratory drives to human genioglossus motoneurons during breathing. Journal of Neurophysiology 95: 2213–2221. 16306175 10.1152/jn.00940.2005

[19] Saboisky JP, Jordan AS, Eckert DJ, White DP, Trinder JA, et al (2010) Recruitment and rate-coding strategies of the human genioglossus muscle. Journal of Applied Physiology 109: 1939–1949. 20947713 10.1152/japplphysiol.00812.2010 PMC3006403

[20] Tsuiki S, Ono T, Ishiwata Y, Kuroda T (2000) Functional divergence of human genioglossus motor units with respiratory-related activity. European Respiratory Journal 15: 906–910. 10853857 10.1034/j.1399-3003.2000.15e16.x

[21] Saboisky JP, Butler JE, Walsh LD, Gandevia SC (2007) New display of the timing and firing frequency of single motor units. Journal of Neuroscience Methods 162: 287–292. 17336391 10.1016/j.jneumeth.2007.01.006

[22] Saboisky JP, Butler JE, McKenzie DK, Gorman RB, Trinder JA, et al (2007) Neural drive to human genioglossus in obstructive sleep apnoea. Journal of Physiology 585: 135–146. 17916615 10.1113/jphysiol.2007.139584 PMC2375464

[23] Sauerland EK, Harper RM (1976) The human tongue during sleep: electromyographic activity of the genioglossus muscle. Experimental Neurology 51: 160–170. 177304 10.1016/0014-4886(76)90061-3

[24] Cheng S, Butler JE, Gandevia SC, Bilston LE (2008) Movement of the tongue during normal breathing in awake healthy humans. Journal of Physiology 586: 4283–4294. 18635645 10.1113/jphysiol.2008.156430 PMC2652195

[25] Hiiemae KM, Palmer JB (2003) Tongue movements in feeding and speech. Critical Reviews in Oral Biology and Medicine 14: 413–429. 14656897 10.1177/154411130301400604

[26] Carrera M, Barbe F, Sauleda J, Tomas M, Gomez C, et al (1999) Patients with obstructive sleep apnea exhibit genioglossus dysfunction that is normalized after treatment with continuous positive airway pressure. American Journal of Respiratory and Critical Care Medicine 159: 1960–1966. 10351945 10.1164/ajrccm.159.6.9809052

[27] Saigusa H, Niimi S, Yamashita K, Gotoh T, Kumada M (2001) Morphological and histochemical studies of the genioglossus muscle. Annals of Otology Rhinology Laryngology 110: 779–784. 10.1177/000348940111000815 11510738

[28] Sacco G, Buchthal F, Rosenfalck P (1962) Motor unit potentials at different ages. Archives of Neurology 6: 366–373. 14495823 10.1001/archneur.1962.00450230028004

[29] Bischoff C, Machetanz J, Conrad B (1991) Is there an age-dependent continuous increase in the duration of the motor unit action potential? Electroencephalogr Clin Neurophysiol 81: 304–311. 1714825 10.1016/0168-5597(91)90017-r

[30] Aniansson A, Grimby G, Hedberg M (1992) Compensatory muscle fiber hypertrophy in elderly men. 73: 812–816. 10.1152/jappl.1992.73.3.812 1400042

[31] Stalberg E, Borges O, Ericsson M, Essen-Gustavsson B, Fawcett PR, et al (1989) The quadriceps femoris muscle in 20–70-year-old subjects: relationship between knee extension torque, electrophysiological parameters, and muscle fiber characteristics. Muscle Nerve 12: 382–389. 2725565 10.1002/mus.880120508

[32] Eastwood PR, Allison GT, Shepherd KL, Szollosi I, Hillman DR (2003) Heterogeneous activity of the human genioglossus muscle assessed by multiple bipolar fine-wire electrodes. Journal of Applied Physiology 94: 1849–1858. 12514165 10.1152/japplphysiol.01017.2002

[33] Jabre JF (1991) Concentric macro electromyography. Muscle & Nerve 14: 820–825. 1922175 10.1002/mus.880140904

[34] Calder KM, Agnew MJ, Stashuk DW, McLean L (2008) Reliability of quantitative EMG analysis of the extensor carpi radialis muscle. Journal of Neuroscience Methods 168: 483–493. 18053579 10.1016/j.jneumeth.2007.10.008

[35] Nashed J, Hamilton-Wright A, Stashuk DW, Faris M, McLean L (2010) Assessing motor deficits in compressive neuropathy using quantitative electromyography. Journal of Neuroengineering and Rehabilitation 7: 39. 20701781 10.1186/1743-0003-7-39 PMC2928769

[36] Stashuk DW (1999) Decomposition and quantitative analysis of clinical electromyographic signals. Medical Engineering & Physics 21: 389–404. 10624736 10.1016/s1350-4533(99)00064-8

[37] Stålberg E, Andreassen S, Falck B, Lang H, Rosenfalck A, et al (1986) Quantitative analysis of individual motor unit potentials: a proposition for standardized terminology and criteria for measurement. Journal of Clinical Neurophysiology 3: 313–348. 3332279 10.1097/00004691-198610000-00003

[38] Nandedkar SD, Barkhaus PE, Sanders DB, Stålberg EV (1988) Analysis of amplitude and area of concentric needle EMG motor unit action potentials. Electroencephalography and Clinical Neurophysiology 69: 561–567. 2453333 10.1016/0013-4694(88)90168-x

[39] Sonoo M (2002) New attempts to quantify concentric needle electromyography. Muscle & Nerve Suppl 11: S98–S102. 10.1002/mus.10154 12116293

[40] Zalewska E, Hausmanowa-Petrusewicz I (1995) Evaluation of MUAP shape irregularity–a new concept of quantification. IEEE transactions on bio-medical engineering 42: 616–620. 7790018 10.1109/10.387201

[41] Zalewska E, Hausmanowa-Petrusewicz I, Stålberg E (2004) Modeling studies on irregular motor unit potentials. Clinical Neurophysiology 115: 543–556. 15036049 10.1016/j.clinph.2003.10.031

[42] Buchthal F, Schmalbruch H (1980) Motor unit of mammalian muscle. Physiological Reviews 60: 90–142. 6766557 10.1152/physrev.1980.60.1.90

[43] Fenrich K, Gordon T (2004) Canadian Association of Neuroscience review: axonal regeneration in the peripheral and central nervous systems–current issues and advances. The Canadian journal of neurological sciences Le journal canadien des sciences neurologiques 31: 142–156. 15198438 10.1017/s0317167100053798

[44] Edwards BA, O’Driscoll DM, Ali A, Jordan AS, Trinder J, et al (2010) Aging and sleep: physiology and pathophysiology. Seminars in Respiratory & Critical Care Medicine 31: 618–633. 20941662 10.1055/s-0030-1265902 PMC3500384

[45] Mehra R, Stone KL, Varosy PD, Hoffman AR, Marcus GM, et al (2009) Nocturnal Arrhythmias across a spectrum of obstructive and central sleep-disordered breathing in older men: outcomes of sleep disorders in older men (MrOS sleep) study. Arch Intern Med 169: 1147–1155. 19546416 10.1001/archinternmed.2009.138 PMC2802061

[46] Hoch CC, Reynolds CF 3rd, Monk TH, Buysse DJ, Yeager AL, et al (1990) Comparison of sleep-disordered breathing among healthy elderly in the seventh, eighth, and ninth decades of life. Sleep 13: 502–511. 2126391 10.1093/sleep/13.6.502

[47] Young T, Palta M, Dempsey J, Skatrud J, Weber S, et al (1993) The occurrence of sleep-disordered breathing among middle-aged adults. New England Journal of Medicine 328: 1230–1235. 8464434 10.1056/NEJM199304293281704

[48] Wellman A, Malhotra A, Jordan AS, Schory K, Gautam S, et al (2007) Chemical control stability in the elderly. Journal of Physiology 581: 291–298. 17317747 10.1113/jphysiol.2006.126409 PMC2075232

[49] Song MK, Ha JH, Ryu SH, Yu J, Park DH (2012) The effect of aging and severity of sleep apnea on heart rate variability indices in obstructive sleep apnea syndrome. Psychiatry Investig 9: 65–72. 10.4306/pi.2012.9.1.65 PMC3285743 22396687

[50] Yamaguchi K, Inoue Y, Ohki N, Satoya N, Inoue F, et al (2014) Gender-specific impacts of apnea, age, and BMI on parasympathetic nerve dysfunction during sleep in patients with obstructive sleep apnea. PLoS One 9: e92808. 24667894 10.1371/journal.pone.0092808 PMC3965452

[51] Goff EA, Nicholas CL, Malaweera AS, Simonds AK, Trinder J, et al (2010) The influence of age on heart rate variability during morning wakefulness. Clin Auton Res 20: 175–182. 19756828 10.1007/s10286-009-0027-0

[52] Eikermann M, Jordan AS, Chamberlin NL, Gautam S, Wellman A, et al (2007) The influence of aging on pharyngeal collapsibility during sleep. Chest 131: 1702–1709. 17413053 10.1378/chest.06-2653 PMC2278166

[53] Goff EA, Nicholas CL, Kleiman J, Spear O, Morrell MJ, et al (2012) The effect of flow limitation on the cardiorespiratory response to arousal from sleep under controlled conditions of chemostimulation in healthy older adults. J Sleep Res 21: 718–723. 22906153 10.1111/j.1365-2869.2012.01019.x

[54] Browne HAK, Adams L, Simonds AK, Morrell MJ (2003) Ageing does not influence the sleep-related decrease in the hypercapnic ventilatory response. European Respiratory Journal 21: 523–529. 12662012 10.1183/09031936.03.00039002

[55] Goff EA, O’Driscoll DM, Simonds AK, Trinder J, Morrell MJ (2008) The cardiovascular response to arousal from sleep decreases with age in healthy adults. Sleep 31: 1009–1017. 18652096 PMC2491511

[56] Morrell MJ, Finn L, McMillan A, Peppard PE (2012) The impact of ageing and sex on the association between sleepiness and sleep disordered breathing. Eur Respir J 40: 386–393. 22241742 10.1183/09031936.00177411 PMC3608395

[57] Malhotra A, Huang Y, Fogel R, Lazic S, Pillar G, et al (2006) Aging influences on pharyngeal anatomy and physiology: the predisposition to pharyngeal collapse. American Journal of Medicine 119: 72 e79–14. 10.1016/j.amjmed.2005.01.077 PMC2287192 16431197

[58] Carlisle T, Carthy ER, Glasser M, Drivas P, McMillan A, et al (2014) Upper airway factors that protect against obstructive sleep apnoea in healthy older males. European Respiratory Journal. 10.1183/09031936.00177213 24833768

[59] Kollias I, Krogstad O (1999) Adult craniocervical and pharyngeal changes–a longitudinal cephalometric study between 22 and 42 years of age. Part I: Morphological craniocervical and hyoid bone changes. Eur J Orthod 21: 333–344. 10502896 10.1093/ejo/21.4.333

[60] Mayer P, Pepin JL, Bettega G, Veale D, Ferretti G, et al (1996) Relationship between body mass index, age and upper airway measurements in snorers and sleep apnoea patients. Eur Respir J 9: 1801–1809. 8880094 10.1183/09031936.96.09091801

[61] Burger CD, Stanson AW, Sheedy PF 2nd, Daniels BK, Shepard JW Jr (1992) Fast-computed tomography evaluation of age-related changes in upper airway structure and function in normal men. Am Rev Respir Dis 145: 846–852. 1554213 10.1164/ajrccm/145.4_Pt_1.846

[62] Martin SE, Mathur R, Marshall I, Douglas NJ (1997) The effect of age, sex, obesity and posture on upper airway size. Eur Respir J 10: 2087–2090. 9311508 10.1183/09031936.97.10092087

[63] Fogel RB, White DP, Pierce RJ, Malhotra A, Edwards JK, et al (2003) Control of upper airway muscle activity in younger versus older men during sleep onset. Journal of Physiology 553: 533–544. 12963804 10.1113/jphysiol.2003.045708 PMC2343562

[64] Worsnop C, Kay A, Kim Y, Trinder J, Pierce R (2000) Effect of age on sleep onset-related changes in respiratory pump and upper airway muscle function. Journal of Applied Physiology 88: 1831–1839. 10797148 10.1152/jappl.2000.88.5.1831

[65] Chowdhuri S, Pierchala L, Aboubakr SE, Shkoukani M, Badr MS (2008) Long-term facilitation of genioglossus activity is present in normal humans during NREM sleep. Respiratory Physiology & Neurobiology 160: 65–75. 17945544 10.1016/j.resp.2007.08.007 PMC2279018

[66] Browne HA, Adams L, Simonds AK, Morrell MJ (2001) Impact of age on breathing and resistive pressure in people with and without sleep apnea. Journal of Applied Physiology 90: 1074–1082. 11181622 10.1152/jappl.2001.90.3.1074

[67] Wilkinson V, Malhotra A, Nicholas CL, Worsnop C, Jordan AS, et al (2008) Discharge patterns of human genioglossus motor units during sleep onset. Sleep 31: 525–533. 18457240 10.1093/sleep/31.4.525 PMC2279758

[68] Nandedkar SD, Stålberg E, Kim Y, Sanders DB, Anne A (1984) Use of signal representation to identify abnormal motor unit potentials in macro EMG. IEEE Transactions on Bio-Medical Engineering 31: 220. 6706351 10.1109/TBME.1984.325332

[69] Nandedkar S, Stålberg E (1983) Simulation of macro EMG motor unit potentials. Electroencephalography and Clinical Neurophysiology 56: 52–62. 6190633 10.1016/0013-4694(83)90006-8

[70] Vogt T, Nix WA, Pfeifer B (1990) Relationship between electrical and mechanical properties of motor units. Journal of Neurology, Neurosurgery, and Psychiatry 53: 331–334. 10.1136/jnnp.53.4.331 PMC1014172 2341847

[71] McComas AJ, Sica RE, Campbell MJ, Upton AR (1971) Functional compensation in partially denervated muscles. Journal of Neurology, Neurosurgery & Psychiatry 34: 453–460. 10.1136/jnnp.34.4.453 PMC493831 4938133

[72] Finsterer J, Fuglsang-Frederiksen A, Mamoli B (1997) Needle EMG of the tongue: motor unit action potential versus peak ratio analysis in limb and bulbar onset amyotrophic lateral sclerosis. Journal of Neurology, Neurosurgery & Psychiatry 63: 175–180. 10.1136/jnnp.63.2.175 PMC2169683 9285455

[73] Martić V, Podnar S (2008) Reference data for quantitative motor unit potential analysis in the genioglossus muscle. Muscle & Nerve 38: 939–940. 18508350 10.1002/mus.21011

[74] Fawcett PR, Johnson MA, Schofield IS (1985) Comparison of electrophysiological and histochemical methods for assessing the spatial distribution of muscle fibres of a motor unit within muscle. Journal of the Neurological Sciences 69: 67–79. 3159855 10.1016/0022-510x(85)90008-5

[75] Buchthal F, Kamieniecka Z (1982) The diagnostic yield of quantified electromyography and quantified muscle biopsy in neuromuscular disorders. Muscle & Nerve 5: 265–280. 7099194 10.1002/mus.880050403

[76] Mayer P, Dematteis M, Pépin JL, Wuyam B, Veale D, et al (1999) Peripheral neuropathy in sleep apnea. A tissue marker of the severity of nocturnal desaturation. American Journal of Respiratory and Critical Care Medicine 159: 213–219. 9872841 10.1164/ajrccm.159.1.9709051

