# Correction: Effects of Aging on Genioglossus Motor Units in Humans

**DOI:** 10.1371/journal.pone.0164252

**Published:** 2016-10-03

**Authors:** Julian P. Saboisky, Daniel W. Stashuk, Andrew Hamilton-Wright, John Trinder, Sanjeev Nandedkar, Atul Malhotra

The legend for Fig 3 appears incorrectly in the published article. Please see the correct Fig 3 and its legend below.

**Fig 3:**
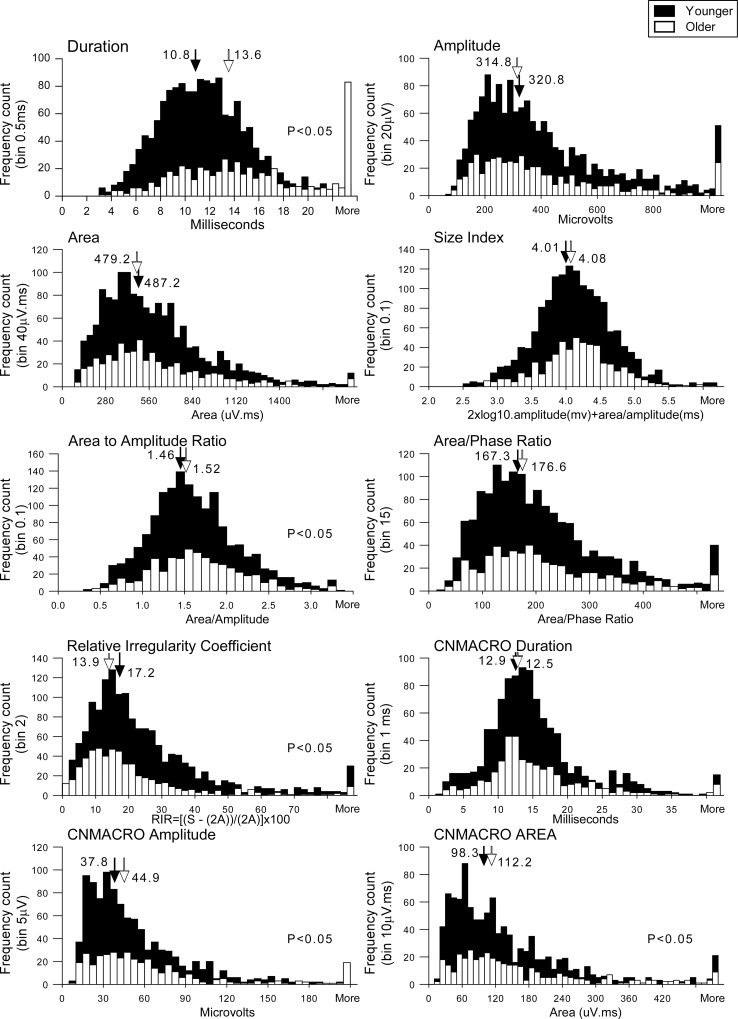
Histogram display with the featured values of selective concentric needle MUPs and concentric needle macro (CNMACRO) MUPs from all 40 subjects. The solid filled columns depict values from younger subjects and the white columns represent values from older subjects. Medians are indicated by arrows in each panel and in each circumstance the filled arrow indicates the median of younger subjects and the unfilled arrow depicts the median of older subjects. Significance is given where appropriate in the respective panels.
